# Scientific output and intensive care units organizational characteristics: a tale of unintended consequences

**DOI:** 10.62675/2965-2774.20250305

**Published:** 2025-01-17

**Authors:** Roberta Muriel Longo Roepke, Juliana Carvalho Ferreira, Alejandro Bruhn

**Affiliations:** 1 Universidade de São Paulo Hospital das Clínicas Faculdade de Medicina São Paulo SP Brazil Trauma and Acute Care Surgery Intensive Care Unit, Hospital das Clínicas, Faculdade de Medicina, Universidade de São Paulo - São Paulo (SP), Brazil.; 2 Universidade de São Paulo Hospital das Clínicas Faculdade de Medicina São Paulo SP Brazil Division of Pulmonology, Instituto do Coração, Hospital das Clínicas, Faculdade de Medicina, Universidade de São Paulo - São Paulo (SP), Brazil.; 3 Pontificia Universidad Católica de Chile Facultad de Medicina Department of Intensive Medicine Santiago Chile Department of Intensive Medicine, Facultad de Medicina, Pontificia Universidad Católica de Chile - Santiago, Chile.

Clinical research in intensive care units (ICUs) is of paramount importance for improving patient care and outcomes. In the era of evidence-based medicine, ensuring that intensive care practices are based on the implementation of new scientific discoveries has become increasingly important. Beyond implementing these advances in medical research, there is evidence from several medical specialties, such as cardiology, surgery and oncology, that active participation in clinical research can improve the quality of care provided to all patients, even those who are not participants in clinical studies.^(1)^ Several mechanisms can contribute to these benefits, including staff training, better adherence to guideline recommendations and other evidence-based practices, all of which can lead to improved patient outcomes.^(2)^ In low- and middle-income countries, despite an increase in research output in recent years, particularly in the field of critical care, there is scarce evidence examining the associations among research output, institutional organizational factors, and patient outcomes.

In this issue of Critical Care Science, Santos et al.^(3)^ presented a secondary analysis of ORganizational CHaractEeriSTics in cRitical cAre (ORCHESTRA),^(4)^ a cohort study that evaluated the associations between critical care organization characteristics and patient outcomes. In this secondary analysis, the authors included data from 93 Brazilian ICUs, examining participation in clinical research in the years 2014 and 2015. They explored the association between an ICU's scientific output and its organizational characteristics on the basis of previous responses to the ORCHESTRA survey. The scientific output of the ICUs was assessed through the number of peer-reviewed publications and their qualifications, assessed as the highest H-index among all publications for each ICU and as the sum of the publications’ Scimago Rank scores. The authors reported an association between an ICU's scientific production and better staffing patterns and process measures. Intensive care units with a greater number of scientific publications and those with publications in journals with an H-index higher than the median had more certified intensivists and a greater number of implemented clinical protocols.

Higher compliance with quality-of-care processes, such as protocol implementation, has been previously shown to be associated with improved patient outcomes in critical care.^(5)^ Does participating in research improve ICU organizational characteristics, or do more qualified and motivated intensivists working in more resourceful and better organized ICUs engage more in clinical research? Alternatively, are other characteristics of the ICUs and their staff be driving the association seen by the authors? The Santos et al.^(3)^ study cannot answer this question because of the cross-sectional design of their analysis; thus, residual confounding and/or reverse causality may explain this association. Even though the observational nature of the study can be seen as a limitation, the observed association can nevertheless be interpreted as an incentive to promote clinical research. Well-organized ICUs, with adequate resources and, most importantly, qualified professionals, may foster work environments where clinical research can thrive and possibly impact patient outcomes.

The alternative explanation for these results is a tale of – mainly positive – unintended consequences. Participating in clinical research in ICUs is a possible pathway for improving patient care, advancing scientific knowledge, and fostering evidence-based practices. Engagement in clinical research may foster an environment of openness to new ideas and protocols, which may lead to a positive ICU culture of change. This ultimately can lead to changes in standard clinical practice, improving the quality of care provided to all patients. Another less desired unintended consequence is the burden that research activities can add to already overburdened healthcare professionals. LMIC researchers, often subject to little or no protected time to conduct research, frequently need to perform research activities as an additional workload. A lack of or insufficient research funding contributes to this issue.

Whatever the directionality of this association, as incentives to improve ICU organizational characteristics are compelling, incentives to increase research output within ICUs may be an important part of better organizational practices. Importantly, these incentives may come from opportunities for career advancement, financial compensation or providing protected time for intensivists to conduct research. By implementing this culture, we may come closer to the ideal scenario of clinical research more closely embedded within clinical practice,^(6)^ which would benefit the healthcare system. We may also provide a potential solution to the increasing prevalence of burnout among intensivists, as clinical research is a nonclinical activity that may bring meaning and joy for intensivists who are enthusiastic about research. Most importantly, fostering clinical research in LMICs could help address research priorities relevant to LMIC communities, increase research diversity and generalizability and improve patient care, the ultimate goal of clinical research.
